# Disturbances of endoplasmic reticulum proteostasis in neurodevelopmental disorders

**DOI:** 10.1042/BST20253035

**Published:** 2025-07-28

**Authors:** Danilo B. Medinas, Nayrob Pereira, Rodolfo Pereira, Giovanna R. da Silva, Victor H.S. Santos

**Affiliations:** 1 Department of Biochemistry, Institute of Chemistry, University of Sao Paulo, Sao Paulo, Brazil; 2 Program of Systems Biology, Institute of Biomedical Sciences, University of Sao Paulo, Sao Paulo, Brazil

**Keywords:** endoplasmic reticulum, genetics, nervous system, neurodevelopmental disorders, proteostasis

## Abstract

The endoplasmic reticulum (ER) is a vital organelle involved in the biogenesis of membrane and secreted proteins. Proteostasis (protein homeostasis) in the ER relies on finely co-ordinated mechanisms for translocation of polypeptides from the cytosol to the organelle lumen and membrane, introduction of co- and post-translational modifications, protein folding and quality control, exportation of mature proteins and disposal of unfolded or aggregated species, besides the regulation of gene expression to adjust the proteostasis network to the cellular demands for protein biogenesis. Neurodevelopmental processes involving neurogenesis, neuronal migration and differentiation, neural circuit wiring, synaptogenesis, among others, require extensive proteome diversification and remodeling, with high fluxes through the secretory pathways constantly challenging ER proteostasis. Genetic defects affecting the different nodes of the ER proteostasis network can severely disturb neurodevelopment. Here, we compile evidence illustrating how perturbations to the different steps of protein biogenesis in the ER can lead to neurological disorders and present major questions to guide research in the field.

## Introduction

According to the International Classification of Diseases 11th Revision (ICD-11) of the World Health Organization (WHO), ‘neurodevelopmental disorders (NDDs) are behavioral and cognitive disorders arising during the developmental period that involve significant difficulties in the acquisition and execution of specific intellectual, motor, language, or social functions [1]. This definition groups together a wide range of neurological problems with onset during infancy, childhood, or adolescence and marked by clinical heterogeneity [2,3]. Intellectual disability (ID), autism spectrum disorders (ASD), and attention deficit hyperactivity disorder (ADHD) are typical examples of such conditions, with psychiatric disorders also being commonly grouped as NDDs [3,4].

The ‘Global report on children with developmental disabilities: from the margins to the mainstream’, a joint document from WHO and United Nations Children’s Emergency Fund (UNICEF), informs 316.8 million affected children and adolescents based on the 2019 Global Burden of Disease study [5,6]. For instance, ASD prevalence in school children of the Western World is ~1.5%, while worldwide figures for ID and ADHD are 2–3% and 5.3%, respectively [7–9]. The socioeconomic impact can be measured in terms of direct medical, direct non-medical, and productivity costs, which, when combined, are estimated to reach US$ 461 billion for ASD only in the USA by the Leigh et al. report [10]. Despite complex and multifactorial etiologies, which can be influenced by poverty, nutritional status, environmental exposure, among others, NDDs have been strongly associated with genetic variants [3].

NDDs can arise from molecular and cellular alterations in different stages of nervous system formation and maturation, often related to neurogenesis, neuronal migration and differentiation, and synaptic activity [2,3]. These processes rely on transcription factors that determine commitment of progenitor cells to neuronal fate and exert spatiotemporal regulation of gene expression profiles that specify neuronal type and promote neural circuit wiring [11–13]. Thus, mutations in genes encoding transcription factors can greatly disturb neurodevelopment, causing diseases [14]. Moreover, genetic mutations affecting basic cellular pathways such as energy metabolism, DNA damage response, protein biogenesis, among others, can perturb specific neuronal populations and neural circuits according to the spatiotemporal expression of mutant genes during development and lead to different pathological outcomes [15].

Perturbations affecting a certain biological process can result in different NDDs with varying degrees of severity, while a particular neurological condition can arise from genetic mutations affecting a multitude of cellular pathways [2,3,16]. The study of pathogenic mechanisms underlying phenotype to genotype relationships can illuminate fundamental aspects of neurodevelopment and constitute the basis for precision medicine approaches. The secretory pathway has emerged as a key cellular component supporting neuronal specification, morphogenesis, and function, being affected in most neurological disorders ranging from neurodegenerative diseases to neurodevelopmental conditions [17–19]. The endoplasmic reticulum (ER) is the port of entry in the secretory pathway where protein synthesis, folding, post-translational modifications, and quality control occur. Here, we will provide a focused review on genetic disturbances of ER proteostasis networks associated with pathological conditions that result from altered development of the nervous system.

## ER proteostasis network and NDDs

**Figure 1:**
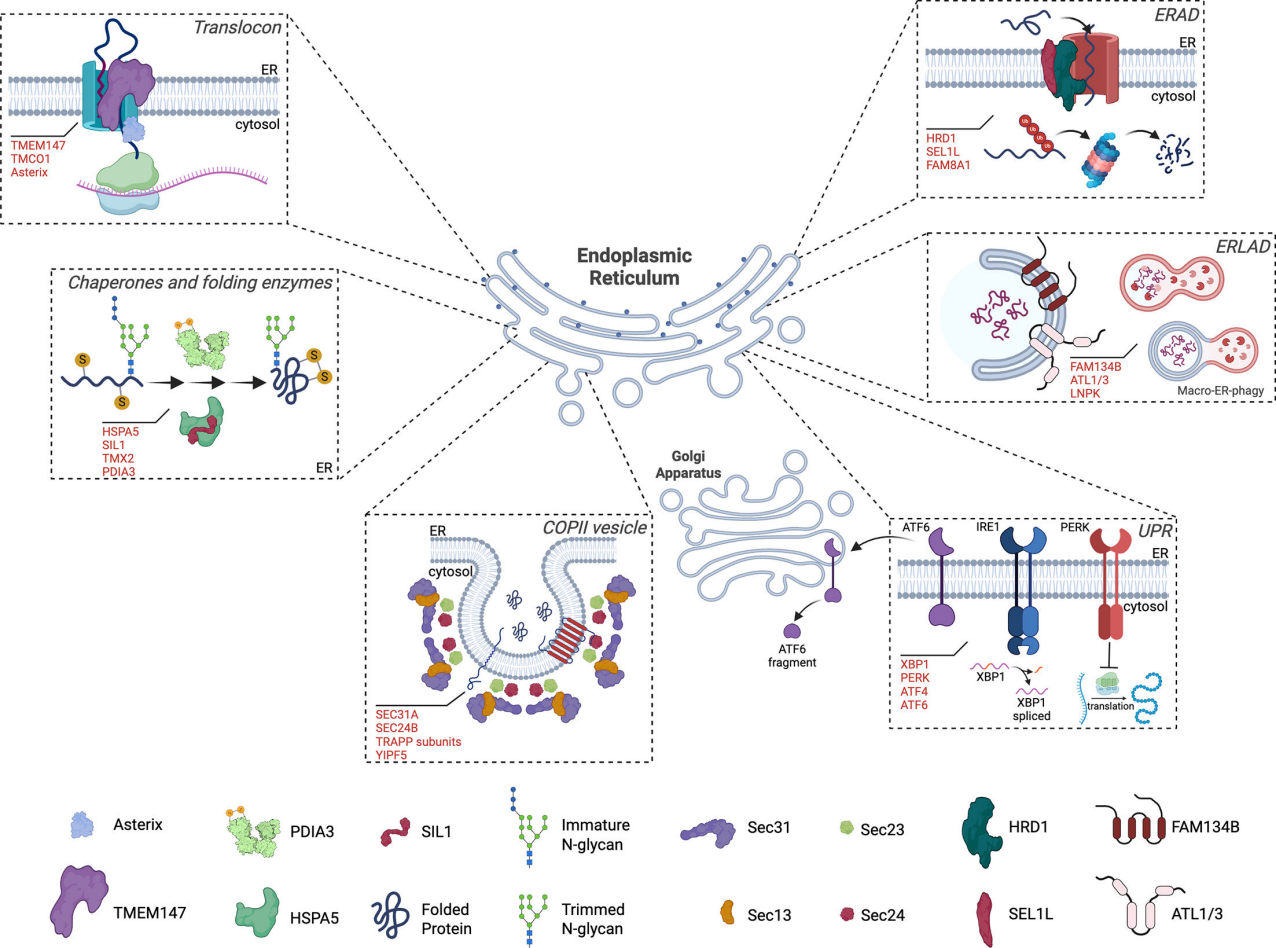
Schematic representation of components of the ER proteostasis affected in neurodevelopmental disorders. The ER proteostasis network can be divided into different nodes, delineated by dashed squares. Representative components associated with neurological problems are illustrated, and the complete list discussed is indicated in red (see also Table 1). ATF4, activating transcription factor 4; ATF6, activating transcription factor 6; ATL1/3, Atlastin 1 and Atlastin 3; COPII, coat protein complex II; ER, endoplasmic reticulum; ERAD, ER-associated degradation; ERLAD, ER-to-lysosome-associated degradation; FAM8A1, family with sequence similarity 8, member A1; FAM134B, family with sequence similarity 134, member B; HRD1, HMG-CoA reductase degradation 1 homolog (E3 ubiquitin ligase); HSPA5, heat shock protein A5; IRE1, inositol-requiring enzyme 1; LNPK, Lunapark; PDIA3, protein disulfide isomerase A3; PERK, protein kinase RNA-activated (PKR)-like ER kinase; SEL1L, adaptor subunit of ERAD E3 ubiquitin ligase; SIL1, nucleotide exchange factor; TMCO1, transmembrane and coiled-coil domains 1; TMEM147, transmembrane protein 147; TMX2, TRX-related transmembrane protein 2; TRAPP, transport protein particle complex; UPR, unfolded protein response; XBP1, X-box binding protein 1; YIPF5, Yip1 Domain Family Member 5. Figure created with biorender.com.

The ER engages several pathways for importing, modifying, and folding polypeptides, in addition to employing mechanisms of protein quality control, disposing of misfolded and aggregated species and sorting folded and mature proteins for exportation to the Golgi apparatus (GA) or their final localization in the cell. Moreover, ER proteostasis is constantly monitored by stress sensors that induce adaptive responses upon increased burden of unfolded or misfolded proteins. We will briefly introduce the major nodes of the ER proteostasis networks and present evidence for their association to pathologies of the nervous system (Figure 1 and Table 1).

**Table 1:** Components of the ER proteostasis network with genetic mutations associated with neurodevelopmental disorders

ER proteostasis node	Gene affected and function^1^	Disease name	Clinical symptoms	Neuropathology	Experimental models	Ref.
**Translocon machinery**	*TMEM147*; translocation of membrane proteins	ND	ID with facial dysmorphism	Brain MRI shows thin corpus callosum for most patients	Patient fibroblasts	[20]
	*TMCO1* (TMCC4); translocation of membrane proteins	ND	ID with facial dysmorphism	Brain MRI shows mild prominence of ventricles	None	[21]
	*WDR83OS* (Asterix); translocon chaperone	ND	ID with hypercholanemia and pruritus	Brain MRI normal for most patients	Zebrafish knockout	[22]
**Chaperones and foldases**	*HSPA5* (GRP78, BiP); chaperone and foldase	Bipolar disorder	Mood alterations	ND	NA	[23]
	*SIL1*; HSPA5 nucleotide exchange factor	Marinesco-Sjogren syndrome	Cerebellar ataxia; cataract; and myopathy	Cerebellar atrophy	NA	[24]
**Redox folding**	*TMX2* (TXNDC14); oxidoreductase	ND	ID with cerebral palsy	Microlissencephaly; cortical polymicrogyria	Patient fibroblasts	[25,26]
	*PDIA3* (GRP58, Erp57); oxidoreductase	ND	ID with facial dysmorphism	Brain MRI normal	AAV-based OE in mouse hippocampus	[27]
**ERAD**	*SYVN1* (HRD1, DER3); ubiquitin ligase	ND	ID with facial dysmorphism; ataxia or hypotonia	Microcephaly	HEK overexpression	[28]
	*SEL1L*; HRD1 interactor	ND	ID with facial dysmorphism; ataxia or hypotonia	Microcephaly	HEK overexpression	[28]
	*FAM8A1* (AHCP); HRD1 complex	Autism spectrum disorder	Available at Simons simplex collection	NA	NA	[29]
**ERLAD**	*RETREG1* (FAM134B); ER-phagy receptor	HSAN II	Impaired nociception; acro-osteolysis of fingers and toes	Axonal sensory neuropathy	RNAi in dorsal root ganglia	[30]
	*ATL1* (SPG3A); GTPase	Hereditary spastic paraplegia	Spasticity; wasting of lower limbs; scoliosis	Abnormal motor evoked potentials	NA	[31]
	*ATL3*; GTPase	HSAN I	Impaired nociception; foot bone destruction	Axonal sensory neuropathy	COS-7 overexpression	[32]
	*LNPK* (Lunapark); membrane curvature stabilizer	ND	Psychomotor delay; ID; hypotonia; epilepsy	Corpus callosum hypoplasia; mild cerebellar hypoplasia and atrophy	Patient fibroblasts	[33]
**ER-Golgi**	*SEC31A* (KIAA0905); COPII component	ND	Spastic quadriplegia; pseudobulbar palsy; epilepsy; deafness	Microcephaly; semilobar holoprosencephaly; agenesis of corpus callosum	Drosophila knockout; CRISPR-KO cell lines	[34]
	*SEC24B*; COPII component	ND	Stillborn	Neural tube defects	KD and OE in zebrafish	[35]
	TRAPPC6B	ND	Epilepsy; hypotonia; autism features	Microcephaly	Zebrafish morphant	[36]
	*TRAPPC12* (TTC-15, GCI-87)	ND	Epilepsy; spasticity; hearing loss	Microcephaly	Patient fibroblasts	[37]
	*YIPF5* (YIP1A, FINGER5)	ND	Epilepsy; diabetes	Microcephaly	KI and KO ESC and patient iPSC	[38]
**UPR**	*XBP1* (TREB5)	Bipolar disorder; schizophrenia; Alzheimer’s disease	NA	NA	NA	[39,40,41]
	*EIF2AK3* (PERK)	Wolcott-Rallison syndrome	ID with diabetes	NA	NA	[42]
	*ATF4* (CREB2)	ND	Cervical dystonia	NA	NA	[43]
	*ATF6*	Achromatopsia	Color blindness; photophobia	Foveal hypoplasia;	KO mice	[44]

1 Protein names are equivalent to gene names and other common protein names are given in parenthesis.

ND, not determined; NA, not available; AAV, adeno-associated virus; KO, knockout; KI, knockin; OE, overexpression; RNAi, interference RNA.

### Importing polypeptides into the ER

The biogenesis of most membrane and secreted proteins in eukaryotes occurs at the ER when the signal recognition particle identifies the signal peptide emerging from ribosomes and induces translational pause for anchoring of ribosomes onto the translocon machinery present in the ER membrane [45]. Then, translation is reinitiated, and the growing polypeptide is translocated through the ER membrane into the organelle lumen or laterally inserted into the membrane [46]. The translocon is a channel composed of several subunits, with the protein Sec61 forming the core allowing the passage of hydrophilic polypeptide segments [47]. Mutations in the genes encoding transmembrane protein 147 (TMEM147), a component of a translocon assembly critical for the biogenesis of membrane multipass proteins such as the glutamate transporter EAAT1 [48], and transmembrane and coiled-coil domains 1 (TMCO1), another translocon component involved in insertion of proteins in the ER membrane, have been reported in ID with facial dysmorphism [20,21]. Moreover, mutations in the genes encoding CCDC47 and Asterix, which form an intramembrane heterodimer participating in the folding of multipass membrane proteins [49], can cause neurodevelopmental problems accompanied by hypercholanemia (augmented serum bile acids) [22].

### Co- and post-translational modifications

Upon entry in the ER lumen, the polypeptide can suffer co-translational modifications, highlighting the process of N-glycosylation of asparagine residues in the consensus sequence Asn-Xxx-Ser/Thr, where Xxx represents any residue except Pro [50]. The process of N-glycosylation confers solubility to proteins and determines protein folding routes and quality control mechanisms in the secretory pathway [50–52]. There is a vast number of rare genetic diseases caused by disruption of glycosylation pathways collectively known as congenital disorders of glycosylation (CDG) [53]. Neurological compromise is a feature of most CDG, which can present varying degrees of severity depending on the biochemical step affected by the genetic mutation (i.e. oligosaccharide assembly, transfer, or trimming). An in-depth review of glycosylation pathways and CDG is beyond the scope here, but available elsewhere [53,54].

Disulfide bond formation is another major co- and post-translational modification occurring in the ER that reflects the redox topology of the cell and extracellular space [55,56]. Oxidizing equivalents are transported into the ER with oxygen consumption by ER oxidases and then transferred to protein disulfide isomerases (PDIs) [57]. PDIs comprise a major family of oxidoreductase in the ER composed of thioredoxin (TRX) domains typically containing the motif CXXC to catalyze disulfide bond formation, reduction, and isomerization, a process that we refer to as redox folding [58]. Over 20 members constitute the PDIs family in humans, which can be subclassified into those containing transmembrane segments (TMX, TRX-related transmembrane proteins) [59,60]. Mutations in the gene encoding TMX2, the only TMX containing the active site facing the cytosol, cause microcephaly accompanied by microlissencephaly, cortical polymicrogyria, and other cell migration defects [25,26]. TMX2 function has been linked to mitochondria–ER contact sites with pathogenic mutations possibly affecting energy metabolism [25]. Recently, we have reported a loss-of-function mutation in protein disulfide isomerase A3 (PDIA3) causing severe ID accompanied by developmental delay with no gross brain abnormalities [27]. Experimental models in cell culture and *in vivo* indicated that mutant PDIA3 may cause neurological problems due to altered neuronal connectivity and synaptic activity [27].

### Protein folding, quality control, and exit to the Golgi Apparatus

BiP [also known as heat shock protein A5 (HSPA5)] is a major chaperone in the ER belonging to the heat shock protein family involved in protein folding, quality control, and regulation of stress responses [52]. Polymorphisms in the *HSPA5* promoter were described as risk factors for bipolar disorder [23]. Moreover, mutations in the gene encoding SIL1, a nucleotide exchange factor for BiP, cause Marinesco-Sjögren syndrome, a disease characterized by cerebellar ataxia and myopathy [24,61]. Disturbing BiP function in mice leads to abnormal formation of neuronal layers of the cerebellum and cerebral cortex due to decreased secretion of reelin, a glycoprotein of the extracellular matrix involved in regulation of neuronal migration and positioning during neurodevelopment [62]. Of note, mutant reelin with reduced secretion has been associated with ASD [63], implying that BiP deficiency may phenocopy molecular aspects of this condition.

Unfolded proteins in the ER are targeted to proteolysis by the ER-associated degradation (ERAD) machinery that retro-translocates the polypeptide from the ER lumen to the cytosol, where it is tagged with ubiquitin for proteasome digestion [64]. Mutations in *SYVN1* and *SEL1*, the genes encoding HMG-CoA reductase degradation 1 homolog (HRD1), an ubiquitin ligase, and SEL1, an HDR1 interactor, can cause NDDs such as ID, ataxia, and facial dysmorphisms by distinct mechanisms, including deficient substrate recruitment, SEL1L-HRD1 complex formation, and HRD1 activity [28]. Moreover, *de novo* mutations in the gene encoding FAM8A1, another HDR1 interactor important for assembly of ERAD machinery [65], have been associated with ASD [29]. In addition to ERAD, selective clearance of portions of the ER containing protein aggregates occurs by lysosomal pathways, collectively called ER-to-lysosome-associated degradation (ERLAD), which can involve the fusion of ER-derived vesicles with lysosomes, as well as the engulfment of ER fragments directly by lysosomes (micro-ER-phagy) or through autophagosomes with subsequent fusion with lysosomes (macro-ER-phagy) [66,67]. ER-phagy relies on receptors localized in the ER membrane that mediate ER fragmentation and delivery to lysosomes, such as the protein family with sequence similarity 134 (FAM134), Atlastins (ATL), Reticulon 3 (RTN3), among others [66,67]. Loss-of-function mutations in the gene encoding FAM134B cause hereditary sensory and autonomic neuropathy type II (HSAN II), marked by impaired nociception [30]. ATL3 is a GTPase acting as ER reshaping factor enriched in three-way junctions that is associated with HSAN I due to a dominant-negative mutation that precludes the protein localization to branch points [32]. Moreover, homozygous loss-of-function mutations in *LNPK*, encoding Lunapark, a protein stabilizing ER curvature within tubular three-way junctions, have been shown to cause ID accompanied by hypotonia, epilepsy, and corpus callosum hypoplasia [33]. On the other hand, dominant mutations in the gene encoding ATL1 cause young-onset spastic paraplegia [31].

Mature proteins exit the ER toward the GA through coated vesicles decorated with coat protein complex II (COPII), while ER proteins are returned from GA by vesicles coated with COPI [68]. The COPII components mediate different steps of vesicle formation, including segregation of ER subdomains, cargo selection, and vesicle shaping [68]. Disruption of their function can result in varied pathological outcomes [69]. For instance, SEC24 is a cargo adaptor, and loss-of-function mutations in its encoding gene can lead to early lethality due to defects in neural tube closure [35], an effect also observed in mice and linked to disruption of the planar cell polarity pathway [70]. SEC13 and SEC31 promote membrane curvature during vesicle formation [68]. Mutations in SEC31 have been associated with a neurodevelopmental syndrome characterized by microcephaly, epilepsy, and premature death [34]. For delivering their cargo to GA, COPII vesicles interact with transport protein particle complex (TRAPP) through SEC23/24, which triggers a cascade of molecular events leading to vesicle fusion with target membranes in the GA [68]. Many mutations in TRAPP subunits have been reported to cause diverse neurodevelopmental problems [69], including ASD features accompanied by neuronal hyperexcitability [36], and encephalopathy associated with delayed ER to GA transport and GA fragmentation [37]. Yip1 Domain Family Member 5 (YIPF5) is a protein involved in anterograde and retrograde ER to GA transport that is localized at ER exit sites, ER-GA intermediate compartments, and *cis*-GA with loss-of-function mutations in its encoding gene causing microcephaly accompanied by neonatal diabetes [38]. YIPF5 deficiency has been associated with retention of proinsulin in the ER of β-cells and apoptosis triggered by ER stress, whereas the impact on neurodevelopment and neuronal function remains to be established [38].

### Stress response to proteostasis imbalance

When misfolded proteins accumulate in the ER, a condition known as ER stress, either as a result of physiological conditions that demand high rates of protein biogenesis in the secretory pathway or because of pathogenic insults of varied sources, signal transduction pathways collectively known as unfolded protein response (UPR) are activated to re-establish ER proteostasis [71]. If the condition is irremediable, the UPR mediates cell death through apoptosis [72]. The signal transduction relies on stress sensors in the ER membrane that detect misfolded proteins in the organelle lumen and execute molecular pathways in the cytosol to attenuate protein translation and reprogram gene expression through specific transcription factors [73]. The three main sensors are inositol-requiring enzyme 1 (IRE1), protein kinase RNA-activated (PKR)-like ER kinase (PERK), and activating transcription factor 6 (ATF6) [73].

IRE1 has RNase activity to degrade specific mRNA and catalyze the unconventional splicing of X-box binding protein 1 (XBP1), leading to generation of the transcription factor XBP1s to promote adaptive gene expression programs [74]. Of note, a polymorphism in the *XBP1* promoter that impairs the positive feedback loop by the transcription factor has been linked to psychiatric conditions such as bipolar disorder [39], schizophrenia [40], among others [17], and Alzheimer’s disease [41]. Additionally, IRE1 serves as scaffolding on the cytosolic face of the ER membrane to assemble different signaling platforms, highlighting its binding to filamin A (FLNA) [75]. FLNA is an actin cross-linking protein essential for cytoskeleton dynamics, and mutations in its encoding gene cause periventricular nodular heterotopia, a neurodevelopmental condition associated with altered cortical neuronal migration, leading to epilepsy and ID [76]. Mutations in the gene encoding RNF13, an IRE1-interacting protein, have been shown to cause an infantile neurodegenerative disorder marked by IRE1-driven stress signaling and increased apoptosis [77].

Upon activation, PERK phosphorylates eukaryotic initiation factor 2 alpha (eIF2α) to arrest translation, contributing to re-establish ER proteostasis by reducing the flux of polypeptides in the organelle [71]. Mutations in *EIF2AK3*, encoding PERK, cause Wolcott-Rallison syndrome, an autosomal recessive neurodevelopmental disease characterized by ID and infancy-onset diabetes [42]. Despite the global decrease in translation, eIF2α phosphorylation triggers the synthesis of selected proteins contributing to the stress response, such as activating transcription factor 4 (ATF4) [72]. ATF4 is a transcription factor that regulates the expression of genes involved in amino acid import and antioxidant response [78,79]. ATF4 activation has been correlated with neurogenesis and its down-regulation with the formation of neuronal layers of the cortex [80,81]. Moreover, ATF4 has been shown to participate in synaptic function and memory formation [82,83]. Mutations that compromise ATF4 function have been described in cervical dystonia, a painful condition marked by motor disability and involuntary movements [43]. ER stress induces translocation of ATF6 for the GA, where it is cleaved to generate ATF6f, a transcription factor that promotes gene expression of ER components, such as chaperones, ERAD machinery, among others [84]. Mutations in the gene for ATF6 cause achromatopsia, a disease characterized by color blindness with reduced visual acuity and absence of cone cells, which are photoreceptor cells making synapses with bipolar neurons [44].

## Concluding remarks and outstanding questions

The ER plays major roles in neuronal morphogenesis and activity, being distributed virtually throughout neurons' perimeter including the soma, axon, and dendrites [85]. The neuronal ER is even referred to as ‘a neuron within a neuron’ given its function in transmitting ion currents through calcium pumping and release [86]. Moreover, the ER promotes lipid synthesis and protein biogenesis, fundamental cellular processes that support plasma membrane expansion necessary for proper neuronal differentiation and that are subject to tight spatiotemporal regulation [87]. In fact, the neuronal architecture and connectivity entail a major challenge for expansion of plasma membrane through the secretory pathway [87]. For instance, the surface area-to-volume ratio in neurons can be hundreds of times higher than in the ‘textbook’ spherical cell, and the growth of neurites (forming the axon and dendrites) implies a much higher expansion rate of surface area compared with volume [87]. Moreover, neurons contain several distinct domains in their plasma membrane, having evolved varied mechanisms for sorting of secretory cargo [88]. While most components of plasma membrane are produced in the soma [87,88], protein synthesis in dendrites and axons seems to ensue from local neuronal demands distally from the soma [89,90]. The transport of secretory cargo over long distances in axons or through highly branched dendritic trees may have exerted temporal constraints for the evolution of alternative routes for protein biogenesis outside the neuronal soma. We highlight differences between the glycosylation profile of the neuronal soma and neuronal processes, the latter containing functional membrane proteins exhibiting unprocessed N-glycosylation typically found in the ER [91]. Such observation suggests the existence of alternative routes for biogenesis and delivery of membrane proteins in neurons, as has been shown for N-methyl-D-aspartate receptors [92]. Moreover, the neuronal proteome is highly complex, with synapses containing over 1000 proteins [93], in addition to being subjected to dynamic remodeling to promote high fidelity wiring of neural circuits and brain function [94,95]. Thus, neuronal development and activity exert a great burden in the ER for protein folding and quality control, and perturbations that impair the delicate balance of the secretory pathway in neurons may result in pathogenicity.

Genetic studies of human diseases can greatly enhance our understanding of gene function and biological processes [96]. Here, we covered major pathways in the ER altered in NDDs due to genetic mutations (Table 1), from polypeptide translocation to protein exportation to the GA, including protein folding and co-/post-translational modifications, elimination of misfolded and aggregated proteins by proteasomal and lysosomal pathways, and signal transduction by the UPR. It is critical to understand how such perturbations to ER proteostasis networks affect the different stages of neurodevelopment involving neurogenesis, neuronal migration and differentiation, neural circuit wiring, synaptogenesis, and synaptic plasticity, among others. Outstanding questions to guide future efforts in the field include: 1) What is the secretory cargo affected in the different NDDs? 2) What are the spatiotemporal gene expression trajectories of mutant ER components associated with these pathologies? Are they related to differential neuronal vulnerability? 3) Do glial cells play any role in pathogenic mechanisms? 4) What are the neurodevelopmental pathways affected by perturbations to ER proteostasis (e.g. specification, migration, connectivity, synaptogenesis)? 5) Can ER proteostasis defects be rescued during embryogenesis or early life? Whether and to which extent other cellular functions are compromised upon disturbances to ER proteostasis in the nervous system also warrants investigation.

**Perspectives**

- Neurodevelopmental disorders (NDDs) encompass a wide range of pathologies, from life-long disabling illnesses to deadly conditions. A significant proportion of cases is caused by genetic mutations, which can be either inherited or somatic. The identification of altered genes greatly enhanced our understanding of neurodevelopment and vulnerability pathways for several neurological symptoms, improving clinical management.
- Endoplasmic reticulum (ER) proteostasis emerged as a key cellular pathway for neurodevelopment that is linked to several NDDs. It has become apparent that the nervous system is particularly sensitive to perturbations in the ER proteostasis network, possibly because the highly complex neuronal architecture and functional domains depend on a finely regulated activity of the secretory pathway.
- Future work needs to be focused on generating a molecular atlas of the ER proteostasis in different brain regions and cellular populations during neurodevelopment to precisely define selective neuronal vulnerability, as well as defects in neural wiring and synaptic plasticity, that lead to malformation and malfunctioning of the nervous system. Such knowledge is essential to further our understanding of NDDs and the development of prognostic tools and therapeutics.

## Abbreviations

ADHD: attention deficit hyperactivity disorder
ASD: autism spectrum disorders
ATF4: activating transcription factor 4
ATF6: activating transcription factor 6
ATL1/3: Atlastin 1 and Atlastin 3
COPII: coat protein complex II
ER: endoplasmic reticulum
ERAD: ER-associated degradation
ERLAD: ER-to-lysosome-associated degradation
FAM8A1: family with sequence similarity 8, member A1
FAM134B: family with sequence similarity 134, member B
FLNA: filamin A
GA: Golgi apparatus
HDR1: HMG-CoA reductase degradation 1 homolog
HSPA5: heat shock protein A5
ID: intellectual disability
IRE1: inositol-requiring enzyme 1
NDDs: neurodevelopmental disorders
PDIA3: protein disulfide isomerase A3
PERK: protein kinase RNA-activated (PKR)-like ER kinase
TRAPP: transport protein particle complex
UPR: unfolded protein response
XBP1: X-box binding protein 1
YIPF5: Yip1 Domain Family Member 5
eIF2α: eukaryotic initiation factor 2 alpha
